# Incidental prostate cancer status in two tertiary centers in Kigali, Rwanda: insights from a retrospective review

**DOI:** 10.11604/pamj.2025.52.124.47310

**Published:** 2025-11-23

**Authors:** Theophile Ndayishimye, Diane Joyeuse Mutuyimana, Sonia Ikugabire, Jean Luc Mwizerwa, Emmanuel Byakagaba, Edouard Ngendahayo, Afrika Guido Gasana, Emmanuel Muhawenimana

**Affiliations:** 1 University of Rwanda, College of Medicine and Health Sciences, Kigali, Rwanda,; 2 Rwanda Military Referral and Teaching Hospital, Kigali, Rwanda,; 3 King Faisal Hospital, Kigali, Rwanda,; 4 Kigali University Teaching Hospital, Kigali, Rwanda

**Keywords:** Incidence of prostate cancer, benign prostate hyperplasia, trans-urethral resection of prostate, open simple prostatectomy, prostate-specific antigen

## Abstract

**Introduction:**

prostate cancer is reported in 16.7% of benign prostate hyperplasia (BPH) surgical specimens. This incidental prostate cancer (IPCa) is usually low grade. While incidence rates vary widely across regions, no published data exist from Rwanda, thus motivating this study.

**Methods:**

this was a retrospective cross-sectional analysis of Trans-Urethral Resection of Prostate (TURP) and open simple prostatectomy specimens from two tertiary hospitals between January 2015 and October 2022 to identify IPCa rate. The clinical characteristics and pathology reports were retrieved. Independent t-test, Fisher´s exact test, and logistic regression were performed to assess associations between clinical characteristics and occurrence of IPCa.

**Results:**

we included 153 patients, mean age 70 years (SD: ±10). 140/153 patients had Lower Urinary Tract Symptoms (LUTS), macrohematuria 4/153, and low back pain 1/153. IPCa was diagnosed in six individuals (6/153, 4%). One patient (1/6) with hematuria and the sole patient with low back pain had IPCa diagnosis (respectively OR: 16.4, 95% CI: 1.1 - 235.9, P=0.04 and OR 38.8, 95% CI: 1.5 - 881.2, P=0.02). Age, prostate volume, and a PSA >4ng/mL were not predictors of IPCa. Grade group (GG) 2 had 2/6 patients, while GG 1, 3, 4, and 5 had 1/6 each. Clinically, 1/6 were cT1a, 5/6 cT1b, with one patient upstaged to T3b postoperatively. Cancer management was watchful waiting in 2/6, active surveillance in 1/6, and androgen deprivation therapy in 3/6 patients.

**Conclusion:**

the incidence of prostate cancer in BPH specimens may be low but occasionally high-grade. Patients with symptoms beyond LUTS need careful assessment preoperatively. Larger prospective studies are needed to corroborate these findings for clinical use.

## Introduction

Prostate cancer is the second most common and fifth leading cause of cancer-related deaths in men [1,2]. In 2018, prostate cancer accounted for 7.1% of worldwide cancer diagnoses in men [1,3] with variable regional incidences. It is the most frequent cancer in Europeans (24%), the second most common in Americans (9.5%) [4,5] and relatively lower rates in East and North Africa [6]. Prostate cancer is concurrent with benign prostate hyperplasia (BPH) in 83%, and incidentally, found in up to 16.7% of Transurethral Resection of Prostate (TURP) specimens [1,7]. Multiple studies have linked IPCa to older age, high Prostate Specific Antigen (PSA), abnormal Digital Rectal Examination (DRE), low prostate volume, and high BMI. Obesity trait masks cancer prior to BPH surgery due to DRE difficulties and PSA dilution effects [1,3,8-14]. Following the widespread use of PSA screening, the rate of IPCa has declined significantly from 27% to 9% [11] but is still reported in high proportions in low-resource settings (21.7%) [8,12,14].

Most IPCa are low-grade and may not require radical surgery or radiotherapy for curative purposes [8]. For example, in China, IPCa was found in 4.7% of cases, with most cases assigned a Gleason score of 3+3=6, indicating low-grade cancer [3]. This rate is similar to 5.6% found by Kizilkan *et al*. (2022) in their retrospective analysis of 430 patients' records [15]. Similarly, a study in Europe identified IPCa in 6.4% of patients, 91% of whom had grade 1 disease [3]. In Pakistan, IPCa among patients who underwent TURP for BPH was 10.7% and 90.9% had low-grade cancer (T1b) [16]. Despite low-risk disease being reported occasionally, intermediate and high risk may occur. For instance, in the analysis of 793 TURP specimens, one patient had a Gleason score of 3 + 4 = 7, and of the IPCa patients, one received radiotherapy, while three underwent radical prostatectomy, challenging procedures due to a prior BPH surgery [1,3,8-10].

In Rwanda, surgery for BPH-mainly TURP and simple prostatectomy-has increased markedly over the past decade, following the launch of Rwanda's first urology training program in 2014. This rise, along with PSA screening, increased the detection of low-risk cancers and reduced unnecessary biopsies. However, ruling out concurrent prostate cancer before BPH surgery remains crucial, as radical prostatectomy or radiotherapy afterward is technically difficult and associated with more complications [2]. To date, no published data exist on the incidence of cancer among Rwandan BPH surgery patients. This study aimed to determine the rate of incidental prostate cancer and identify preoperative predictors in two tertiary hospitals.

## Methods

**Study design and setting**: this was a cross-sectional retrospective study of prostate specimens obtained during benign prostatic surgery conducted between January 2015 and October 2022 at the University Teaching Hospital of Kigali (CHUK) and King Faisal Hospital (KFH) Kigali, Rwanda, both tertiary referral hospitals and pioneer centers for BPH surgery in Kigali, Rwanda.

**Study population:** the study included 153 men who underwent transurethral resection of the prostate (TURP) or open simple prostatectomy for presumed benign prostatic hyperplasia (BPH) within the study period. Patients with a known prostate cancer diagnosis before surgery (n=21), incomplete medical records (n=42), or non-detailed histopathology reports (n=11) were excluded.

**Data collection:** data were obtained from electronic medical records (EMR), theatre registries, and printed files using a predesigned questionnaire capturing socio-demographic, clinical, and histopathological details. PSA and prostate ultrasound results from the study hospitals were included, while results from outside facilities were excluded unless performed at one of the study sites.

**Definitions:** the primary outcome was incidental prostate cancer (IPCa), confirmed by histological examination of surgical specimens within each hospital's pathology laboratory. Variables analyzed included age, PSA levels, PSA density, prostate size, DRE findings, surgical technique, Gleason score, and subsequent management. Quantitative variables such as age and PSA were analyzed as both continuous and categorical (<65 vs >65 years; <4 ng/ml vs >4 ng/ml). PSA density (<0.15 vs >0.15) and prostate size (<40g vs. >40g) were also categorized, while DRE nodules were treated as binary (yes/no).

**Statistical analysis**: data were analyzed using SPSS version 25.0 (IBM Corp., 2017). Continuous variables were expressed as means ± standard deviation, while categorical variables were presented as frequencies and percentages. Fisher´s exact test and independent sample t-tests were applied for group comparisons. Logistic regression analyses were used to identify predictors of incidental prostate cancer, with statistical significance set at p <0.05 and a 95% confidence interval.

**Ethical considerations:** ethical approval was obtained from the institutional review boards of both hospitals (KFH approval No. KFH/2022/031/IRB; CHUK approval No. EC/CHUK/149/2022).

## Results

**Figure 1:**
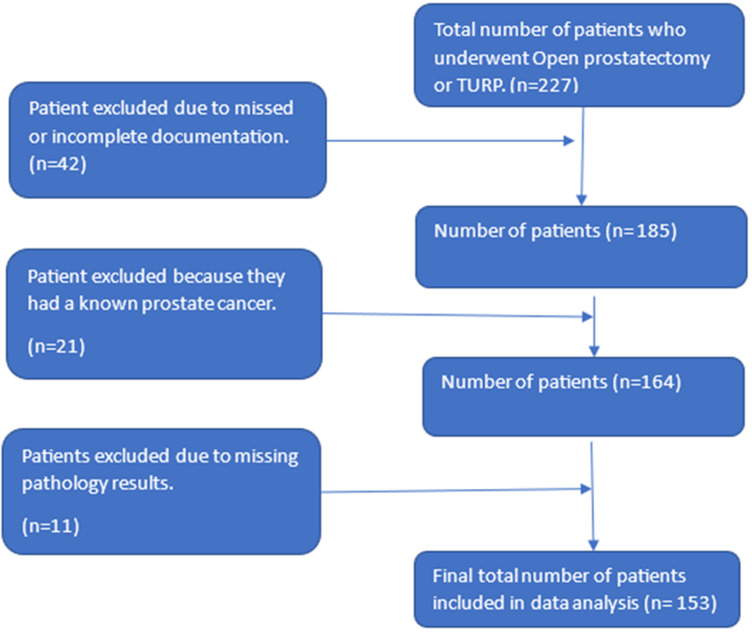
selection of participants from prostatectomy and Transurethral Resection of Prostate at Centre Hospitalier Universitaire de Kigali and King Faisal Hospital, Kigali, Rwanda (Jan 2015-Oct 2022; N=227) (final cohort: 153 after exclusions)

**Cohort characteristics:** we recorded 227 eligible patients from theatre registries who had undergone TURP or open simple prostatectomy at either King Faisal Hospital (KFH) or University Teaching Hospital of Kigali (CHUK) during our study period. We had 74 patients excluded due to incomplete medical records (n=42), a known cancer diagnosis before surgery (n=21), or missing complete histopathology reports (n=11). Our final analysis included 153 patients (Figure 1) with a mean age of 70 years (SD: ± 10), younger than 65 years (49/153) and older than 65 years (104/153). Most of the patients (140/153, 95.3%) presented with lower urinary tract symptoms (LUTS), 4/153 with hematuria and 1/153 with low back pain (Table 1). Average prostate size on ultrasound was 46ml (IQR = 36 - 80) for non-cancer patients and 72ml (IQR = 41 - 99) for the IPCa patients (P=0.5), while average PSA was 5.6ng/ml (IQR = 2.8 - 9) for non-cancer and 9.9ng/ml (IQR = 6 - 28) for the IPCa patients (P=0.9). Regarding pre-operative biopsy, 79.2% (n=121) did not undergo a pre-operative prostate biopsy, while 20.8% (n=32) did. A biopsy was done due to isolated high PSA in 8/32, raised PSA (>4ng/ml) with abnormal DRE in 4/32, and solely abnormal DRE in 20/32. The common reported histology on pre-operative biopsy was BPH in 29/32 alone or combined with prostatitis in 7/32. TURP was done in 71% (109/153) and open simple prostatectomy in 29% (45/153).

**Table 1:** clinical characteristics of participants stratified by incidental prostate cancer status

	Incidental prostate cancer		
	No (N=147)	Yes (N=6)	
	N (%)	N (%)	P value
**Age group**			
≤ 65	49 (33.3)	0 (0.0)	0.178
> 65	98 (66.7)	6 (100.0)	
**Mean age: 70.2 ± 10 years**			
**LUTS**			
No	7 (4.7)	1 (16.7)	0.279
Yes	140 (95.3)	5 (83.3)	
**Hematuria**			
No	144 (98)	5 (83.3)	0.149
Yes	3 (2)	1 (16.7)	
**Low back pain**			
No	146 (99.4)	5 (83.3)	0.077
Yes	1 (0.6)	1 (16.7)	
**Acute urinary retention**			
No	124 (84.3)	4 (66.7)	0.254
Yes	23 (15.7)	2 (33.3)	
**PSA level**			
≤ 4 ng/mL	54 (36.8)	0 (0.0)	
> 4 ng/mL	93 (63.2)	6 (100.0)	0.09
**Prostate size on ultrasound**			
≤ 40 g	40 (39.6)	1 (25)	
> 40 g	61 (60.4)	3 (75)	1
**PSA density**			
≤ 0.15	97 (66)	2 (33.3)	
> 0.15	50 (34)	4 (66.7)	0.186
**Prostate biopsy prior to surgery**			
No	118 (80.3)	4 (66.7)	
Yes	29 (19.7	2 (33.3)	0.602

BPH: benign prostate hyperplasia; CHUK: Centre Hospitalier Universitaire de Kigali; KFH: King Faisal Hospital; LUTS: lower urinary tract symptoms; PSA: prostate-specific antigen. Note. P values were obtained using Fisher's exact test

**Incidental prostate cancer and predictors:** IPCa was diagnosed in 6/153 (4%). All IPCa patients were older than 65 years (P=0.9), and 5/6 had LUTS (P=0.23), one patient (1/6) had hematuria (P=0.068), 1/6 had low back pain (P=0.02) (Table 1). Cancer was diagnosed in 2/101 patients with normal DRE versus 4/52 with abnormal DRE (P=0.1) and 2/6 with the presence of nodules (P=0.2) (Table 2). Also, prostate volume did not correlate with cancer detection (P=0.5).

**Table 2:** digital rectal examination (DRE) findings on the patient's assessment before surgery

	Incidental prostate cancer				
	No		Yes		
	N (%)	Mean ± SD	N (%)	Mean± SD	P Value
**DRE**					
Normal	99 (67.3)		2 (33.3)		
Abnormal	48 (32.7)		4 (66.7)		0.109
**Consistency on DRE**					
Smooth	105 (71.4)		3 (50.0)		
Hard	31 (21.1)		2 (33.3)		
Induration	11 (7.5)		1 (16.7)		
**Nodules on DRE**					
No	127 (86.4)		4 (66.7)		0.199
Yes	20 (13.6)		2 (33.3)		

DRE: digital rectal exam; SD: standard deviation

On the univariate logistic regression, low back pain and hematuria reached significance with P<0.05: OR 29.2, 95% CI: 1.588 - 536.887, P=0.023, and OR 9.6, 95% CI: 0.843 - 109.315, P=0.068, respectively, but the latter was borderline. Abnormal DRE (OR 4.125, 95% CI: 0.730 - 23.315, P=0.109) and PSA density (OR 3.9, 95% CI: 0.687 - 21.915, P=0.12) showed weak associations but did not reach conventional thresholds, likely due to small sample size. The age, lower urinary tract symptoms (LUTS), acute urinary retention presentation, and prostate size on ultrasound were not significant (Table 3). In multivariate analysis, hematuria and low back pain were independent predictors of incidental prostate cancer (OR 16.407, 95% CI: 1.141 - 235.928, P =0.040, and low back pain: OR 38.838, 95% CI: 1.540 - 881.220, P=0.026). These associations were observed with a small number of cases, which are likely to explain the inflated odds ratio (Table 3).

**Table 3:** univariate and multivariate logistic regression analysis of factors associated with incidental prostate cancer

	Unadjusted OR (95% CI)	P value	Adjusted OR (95% CI)	P value
**Age group (≤ 65)**				
> 65	0 -	0.997		
**Symptoms**				
LUTS	0.250 (0.026 - 2.438)	0.233		
Hematuria	9.6 (0.843 - 109.315)	0.068	16.407 (1.141 - 235.928)	0.04
Low back pain	29.2 (1.588 - 536.887)	0.023	38.838 (1.540 - 881.220)	0.026
Acute urinary retention	2.696 (0.466 - 15.586)	0.268		
**DRE-consistency normal**				
Abnormal	4.125 (0.730 - 23.315)	0.109		
**Nodules in DRE (no)**				
Yes	3.175 (0.545 - 18.495)	0.199		
**PSA - preop (≤ 4ng)**				
> 4ng	0 -	0.997		
**Prostate size/US (≤ 40g)**				
> 40 g	1.967 (0.198 - 19.583)	0.564		
**PSA density (≤ 0.15)**				
> 0.15	3.880 (0.687 - 21.915)	0.125		

95% CI: 95% confidence interval; DRE: digital rectal examination; OR: odds ratio; PSA: prostate-specific antigen; SD: standard deviation; US: ultrasound

**Prostate cancer T staging and management:** prostate cancer histology was exclusively adenocarcinoma, and the tumor volume ranged between 10% and 60% with 1/6 presenting with perineural invasion (PNI). With regards to staging, one patient (1/6) had T1a while 5/6 had T1b disease. Based on International Society of UroPathologist (ISUP) grade groups (GG), group 2 accounted for 2/6 patients while GG 1, 3, 4, and 5 accounted for 1/6 patient each, indicating a mixture of low-grade (16.7%), intermediate (33.3%), and high-grade cancer (50% i.e.: GG 3, 4, and 5). After IPCa diagnosis, one patient was upstaged to T3b on MRI. Management included watchful waiting in 2/6, Active surveillance in 1/6, and ADT for one elderly patient who developed symptoms related to cancer and two other patients with high-grade disease (Table 4).

**Table 4:** clinico-pathological features of incidental prostate cancer cases and subsequent management

S/N	PSA	Gleason	Stage	Volume in %	Type of cancer	PNI	Management
Case 1	9.8	3+3=6	T1b	20%	Prostatic acinar adenocarcinoma	No	Active surveillance
Case 2	35	3+4=7	T1a	NA	Prostate adenocarcinoma	Yes	Watchful waiting
Case 3	5.6	3+4=7	T1b	10%	Prostate adenocarcinoma	No	Watchful waiting
Case 4	9.1	4+4=8	T1b	20%	Invasive acinar adenocarcinoma	No	ADT
Case 5	28	4+3=7	T1b	60%	Prostate adenocarcinoma	No	ADT
Case 6	10.9	4+5=9	T1b upstaged to T3bN1M0	40%	Acinar adenocarcinoma	No	ADT

ADT: androgen deprivation therapy; NA: not available, but the pathologist reported T1a; PNI: perineural invasion

## Discussion

Our study aimed to determine the incidence of prostate cancer in BPH specimens at the University Teaching Hospital of Kigali (CHUK) and King Faisal Hospital in Kigali, Rwanda. We detected 4% of specimens to bear prostate cancer. Preoperative macrohematuria and low back pain were potential independent predictors of incidental prostate cancer. Most patients had a grade group 2 and above, indicating the presence of intermediate and high-grade cancer in need of intervention after diagnosis and stratification.

Our observed incidence of incidental prostate cancer (IPCa) at 4% is comparable to reports from China (4.7%) and Turkey (5.6%) [3,15], but remains lower than those from Pakistan (10.7%), Tombal *et al*. (9%), and Mai *et al*. (8%) [11,12,16,17]. Previous studies identified advanced age, abnormal DRE, elevated PSA, higher PSA density, and smaller prostate volume as key predictors of IPCa [3,9,11,12,17-19]. In our cohort, all IPCa cases occurred in patients older than 65 years with PSA >4 ng/mL, although no significant association was found between IPCa and PSA, PSA density, prostate volume, and abnormal DRE. This may relate to preoperative factors such as long-term indwelling catheters, which can elevate PSA levels in both non-cancer and cancer patients, and low sensitivity of DRE, which only identifies cancer in 4 out of 10 cases [14,20]. Similar to Gunda *et al*. (2018), who found IPCa linked to age and PSA >10 ng/mL [14], and Sakamoto *et al*. (2014), who identified age >75 years as a key predictor [21], our results reinforce the role of age and PSA elevation in risk stratification before BPH surgery.

In a systematic review and meta-analysis by Hansen *et al*. (2022), macrohematuria was identified as a presenting symptom of prostate cancer in up to 36.4% [22], while low back pain is recognized as a sign of locally advanced or metastatic prostate cancer. This may explain our findings of higher grades of cancer in our cohort, where only one patient was a low grade, while others were grade group 2 and above, compared with low-grade cancers reported by previous studies [3,14]. However, comparable to our findings of 50% high-grade cancer are the results of Mohamed *et al*. (2022), who reported a high-grade incidental prostate cancer in 42.2% of the studied cohort in Somalia [8]. Low-grade prostate cancers reported in other reviews were not particularly concerning to the authors, as few required intervention; most were managed with active surveillance due to their favorable long-term survival and similar outcomes compared to those who underwent invasive treatments [3,14]. Elkoushy *et al*. (2015) demonstrated that the survival rates of patients with incidental prostate cancer at 5 and 10 years were 72.8% and 63.5%, respectively, by means of active surveillance [23], however, Guo *et al*. (2022) reported that radiotherapy is also safe for patients with a history of prostate resection, but undergoing open prostatectomy is technically challenging for this population [3] favoring active surveillance or radiotherapy in his assertions. Our findings of higher-grade cancers support these assertions on the importance of more targeted preoperative screening, particularly for patients presenting with advanced cancer symptoms beyond LUTS, those over 65 years of age, or with PSA levels exceeding 4ng/mL.

These findings are clinically significant, particularly in resource-limited settings, indicating that 4% with high-risk cancer cases could be missed in patients undergoing surgery for presumed BPH if histological evaluation of postop specimens is omitted. Clinicians should take a thorough history and prioritize targeted screening, especially in older patients, with symptoms beyond LUTS or elevated PSA, to prevent incidental detection of high-grade tumors that are more difficult to manage post-BPH surgery.

This study was designed to determine the rate of incidental prostate cancer (IPCa) in two tertiary hospitals-the first report of its kind from Rwanda. While its retrospective, cross-sectional design based on EMR data presents inherent limitations such as potential biases of incomplete records and data inconsistencies, we mitigated these by cross-checking all available sources for accuracy and completeness. Although the study does not represent the entire country, it offers valuable insights into the local burden of IPCa and establishes a foundation for future prospective studies to guide national policy and clinical practice.

## Conclusion

Incidental prostate cancer among presumed benign prostatic hyperplasia cases in these tertiary hospitals is uncommon but often high risk, with no strong predictive factors identified. Men over 65 years or those presenting with hematuria, low back pain, or PSA >4 ng/mL should be carefully evaluated and considered for biopsy when it may alter management. The detection of high Gleason scores underscores the need for routine histopathologic review of surgical specimens. Given the study´s retrospective design and limited geographic scope, broader nationwide prospective research is recommended to better define preoperative predictors and guide histopathologic evaluation practices in Rwanda.

### What is known about this topic

- Incidental prostate cancer shows variable incidence across populations and has been modestly reported in resource-constrained countries;
- Management is challenging, with inconsistent guidelines across clinical settings;
- Experts highlight technical difficulties with surgery post-BPH treatment and the toxicity risks of radiotherapy.

### What this study adds

- This study is the first of its kind in Rwanda, to the best of our knowledge, offering insights relevant to local and similar healthcare settings;
- It establishes a low baseline incidence of incidental prostate cancer, which may increase with the current trend of prostate enucleation techniques posing new challenges in already resource-limited systems;
- In contrast with previous literature, this study shows a higher than expected proportion of high-risk tumors.
